# Treatment of Acne Vulgaris With a 650‐ms, 1064‐nm Nd:YAG Laser: A Retrospective Study

**DOI:** 10.1111/jocd.16711

**Published:** 2024-12-12

**Authors:** Idowu D. Olugbade, Anna C. Petty, Joyce Imahiyerobo‐Ip

**Affiliations:** ^1^ The Warren Alpert Medical School of Brown University Providence Rhode Island USA; ^2^ Vibrant Dermatology Dedham Massachusetts USA

**Keywords:** Caucasian, pulse width, skin of color

## Abstract

**Background:**

The 650‐ms, 1064‐nm Nd:YAG laser device may provide superior efficacy and tolerability for the treatment of acne vulgaris over conventional treatments.

**Aim:**

To evaluate the efficacy and tolerability of a 650‐ms laser for the treatment of mild to severe facial acne vulgaris.

**Patients/Methods:**

Records of 225 subjects with mild to severe facial acne vulgaris and treated with a 650‐ms laser were reviewed.

**Results:**

Subjects required a median of 3 treatments to achieve clearance. Clearance was achieved in 108/225 (48%) subjects. Adverse effects were limited to acne flare‐ups and dryness. Treatment with isotretinoin was not required in 180/209 (80%) of subjects. A variety of topical and oral medications and non‐laser procedures may be used in conjunction with the 650‐ms laser without adverse effects. At the 6‐month follow‐up visit, the median Investigator Global Scale (IGA) score was 1.0 (almost clear). For most IGA‐rated parameters differences between white patients and patients with skin of color were not statistically significant.

**Conclusion:**

The 650‐ms, 1064‐nm Nd:YAG laser provides a safe and efficacious treatment of mild to severe acne in patients with white skin and skin of color.

## 1 Introduction

Acne vulgaris is a chronic inflammatory skin disorder that affects approximately 9.4% of the global population, [1]. The pathogenesis of acne involves dysseborrhea and hyperseborrhea, changes in the hyperkeratinization of the pilosebaceous duct, the presence of *Cutibacterium acnes* (*C. acnes*), inflammation, and hormones (including androgens, insulin, and insulin‐like growth factor‐1) [2]. A variety of psychosocial and psychiatric comorbidities associated with acne have also been described [1, 3].

Recent guidelines for managing acne vulgaris [4] strongly recommend benzoyl peroxide, topical retinoids, topical antibiotics, and oral doxycycline. For severe acne or cases unresponsive to standard treatments, oral isotretinoin is advised. Combining topical therapies with different mechanisms, reducing reliance on systemic antibiotics, and incorporating intralesional corticosteroid injections when appropriate are effective strategies for effective acne management. For physical modalities, which include laser and light‐based therapies, evidence is insufficient to develop guidelines.

Topical and oral agents often have a slow onset of action, side effects that can reduce patient compliance, limited efficacy for some individuals, and the risk of antibiotic resistance [5]. Additionally, isotretinoin's teratogenicity poses significant concerns [6]. Due to these challenges, energy‐based devices have gained popularity for managing acne and its comorbidities. The effectiveness of these or any alternative treatments depends on their demonstrated efficacy, safety profile, and potential for high patient compliance.

The present study retrospectively evaluates the efficacy and tolerability of the 650‐ms, 1064‐nm Nd:YAG laser therapy for mild to severe facial acne vulgaris in 225 patients with white and skin of color.

## 2 Materials and Methods

### 2.1 Study Design

Electronic medical records of patients (*n* = 225, aged 14–61 years) diagnosed with mild to severe facial acne vulgaris were reviewed. All patients had been treated with the 650‐ms, 1064‐nm Nd:YAG laser (Neo Elite, Aerolase Corp., Tarrytown, NY). The authors analyzed the number of treatments to achieve clearance as defined by the Investigator's Global Assessment (IGA) scale, adverse events, medications, and other non‐laser procedures used in conjunction with the laser.

### 2.2 Acne Severity Determination

Acne severity was assessed at each visit using the IGA scale in which 0 = clear skin, 1 = almost clear, 2 = mild, 3 = moderate, and 4 = severe, and 5 = very severe. Patients were assigned an initial IGA score from the clinical photos taken immediately before the laser procedure. Each patient's IGA score was independently reviewed and unanimously between at least two investigators.

### 2.3 Procedure

Each patient's face was cleansed with a gentle cleanser and water and patted dry. Frontal, left, and right profile photographs of each patient were taken after each visit prior to laser treatment using an iPad (9.7 in., 2017/2018 Model, 5th/6th Generation. Apple Inc., Cupertino, CA). All procedures were performed without anesthetic or precooling. Patient eyes were protected using sealed eye shields and protective eyewear was worn by those in the treatment room.

### 2.4 Laser Treatments

Laser settings were adjusted on the basis of Fitzpatrick skin type (Tables 1 and 2) to achieve the desired skin response to each laser pulse and to minimize the risk of burns, blisters, and erythema. Lower energies were used on patients with darker skin types.

**TABLE 1:** Step 1 laser settings.

Fitzpatrick skin type	Lens spot size (mm)	Pulse duration (ms)	Energy mode	Fluence (J/cm^2^)
I–III	6	0.65	6	21
IV	6	0.65	5	18
V	6	0.65	4	14
VI	6	0.65	3	11

**TABLE 2:** Step 2 laser settings.

Fitzpatrick skin type	Lens spot size (mm)	Pulse duration (ms)	Energy mode	Fluence (J/cm^2^)
I–III	6	1.5	8	28
IV	6	1.5	6	21
V	6	1.5	5	18
VI	6	1.5	4	11

Laser treatments were performed in two steps (Tables 1 and 2). The treatment beam was applied to affected areas in a grid‐like pattern with 6 passes using the 650‐ms pulse width followed by 2 passes at 1500‐ms pulse duration, thus addressing post‐inflammatory erythema and stimulating collagen remodeling. Use of the longer pulse permitted treatment of post‐inflammatory erythema and hyperpigmentation in all patients irrespective of skin color. Moisturizer and mineral‐based sunscreen were applied immediately after laser treatment. Patients resumed their topical and oral medications, including topical retinoids.

In each treatment, the clinician monitored the heat sensation of the patient and adjusted the energy modes accordingly. For the most effective results; however, the clinician treated each subject with the highest energy mode possible for the skin type.

### 2.5 Data Analysis

Subjects were categorized into two groups: white (Caucasian) and skin of color (African‐American, Hispanic, Asian, and other races). The two groups were compared for the number of treatments to clearance, IGA scores at baseline and at 6 months, adverse effects, and the need for isotretinoin. The numbers for each parameter were small whole numbers with a short range, so a normal distribution of data was not assumed. Instead, a nonparametric Wilcoxon signed rank test was used for comparisons. In cases where the response was binary (yes or no), values of 1 and 0 denoted yes and no, respectively. Statistical analyses were conducted using Analyse‐it Software, Leeds, U.K.

## 3 Results

### 3.1 Patient Demographics and Baseline Characteristics

A total of 255 patients completed one or more treatments. Among these, 89% self‐identified as females and 11% as males. The majority of patients were Caucasian (41%) and African American (38%), while the remaining patients identified as Asian (9%), Hispanic (3%), and other (9%).

#### 3.1.1 Number of Treatments and IGA Score

**FIGURE 1:**
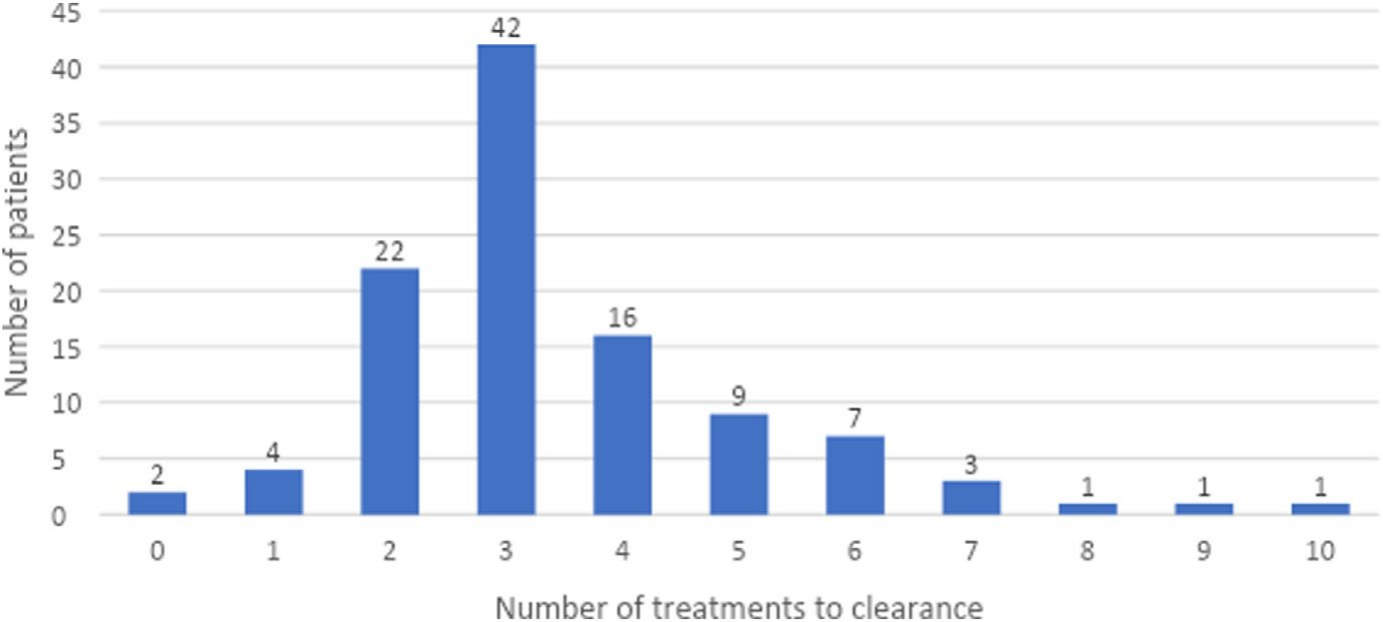
Number of treatments to clearance vs. number of subjects.

As shown in Figure 1, patients required a median of 3 treatments to achieve clearance. Clearance was achieved in 108/225 (48%) patients.

**FIGURE 2:**
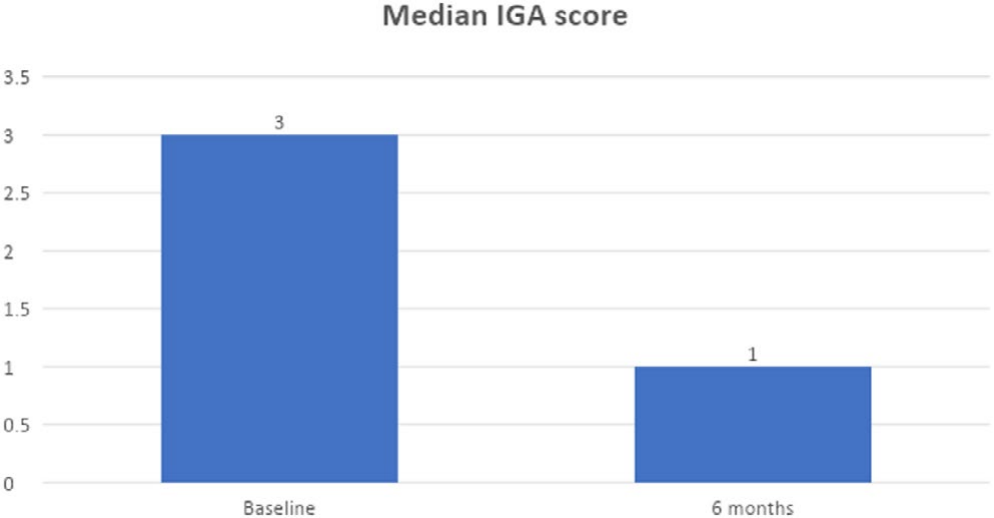
Acne classification (median investigator global assessment [IGA] score) at baseline and 6 months.

Patients were evaluated 6 months after the laser procedure. Figure 2 shows the median IGA scores at baseline and at 6 months. The median score at baseline, 3, denoted moderate severity while the 6‐month score was 1, or almost clear skin.

#### 3.1.2 Adjunctive Therapies

Treatment with isotretinoin was not required in 180/209 (80%) of patients (Table 3). As shown in Table 4, a variety of topical medications were used in conjunction with the 650‐ms laser. The most frequently employed were benzoyl peroxide (BPO), adapalene, azelaic acid, and minocycline. Oral medications are presented in Table 5, with 56.4% of patients requiring antibiotics and 17.3% requiring hormone‐modulating therapies. When a patient achieved clearance, oral medications were discontinued. Additionally, some patients had a variety of non‐laser procedures, primarily chemical peels and extractions (Table 6). The use of adjunctive therapies with the laser was well tolerated by patients and no adverse effects were observed.

**TABLE 3:** Subjects that required isotretinoin.

Yes (%)	No (%)	Recommended (%)	No response (%)
17 (7.5)	180 (80)	4 (1.8)	24 (10.7)

**TABLE 4:** Topical medications used in conjunction with 650‐ms, 1064‐nm Nd:YAG laser.

Topical medication	Total no. of times used in study
Benzoyl peroxide (BPO)	265
Adapalene	125
Azelaic acid	77
Minocycline	52
Tretinoin	25
Trifarotene	23
Hydroquinone	20
Tazarotene	19
Sulfacetamide sodium	18
Tacrolimus	17
Clindamycin	13

**TABLE 5:** Number of subjects requiring oral medications.

Antibiotics (25–150 mg) (%)^a^	Hormone‐modulating therapies (%)^b^	No oral medications (%)
127 (56.4)	39 (17.3)	59 (26.2)

^a^ Doxycycline, seracycline, minocycline.

^b^ Spironolactone, tri‐sprintec, ortho tri‐cyclin.

**TABLE 6:** Non‐laser procedures used in conjunction with 650‐ms, 1064‐nm Nd:YAG laser.

Procedure	Total no. of subjects that received procedure
Chemical peels	87
Extractions	14
SilkPeel MD SP3, dermal infusion	3
Blue light	1

#### 3.1.3 Adverse Events Assessment

Serious adverse events were not observed during the study. Non‐serious adverse effects were recorded for 210 patients and broken down into 5 categories (Table 7). The most common adverse effects were acne flare‐up (55.7%) and dryness (13.3%). The rest were limited to erythema (1.9%), edema (0.5%), and itchiness (1%).

**TABLE 7:** Adverse effects associated with the 650‐ms, 1064‐nm Nd:YAG laser.

Adverse effect	Response (%)		
	No	Yes	Respondents
Erythema	206 (98.1)	4 (1.9)	210
Edema	209 (99.5)	1 (0.5)	210
Acne flare‐up	93 (44.3)	117 (55.7)	210
Dryness	182 (86.7)	28 (13.3)	210
Itchiness	208 (99.0)	2 (1.0)	210

#### 3.1.4 Treatment of White and Skin of Color Patients

As shown in Table 8, significant differences were not observed for the number of treatments to clearance, IGA scores at baseline and 6 months, need for isotretinoin, or adverse effects between white skin and skin of color.

**TABLE 8:** Comparisons between skin of color (SOC) and white skin subjects using Wilcoxon signed rank test.

Parameter	Median (min, max)		*p*
	SOC	White	
No. Tx to clearance	3.0 (0, 10)	3.0 (0, 8)	0.7052
IGA			
Baseline	3.0 (0, 4)	3.0 (0, 4)	0.8098
6 months	2.0 (0, 3)	1.0 (0, 4)	0.8068
Need isotretinoin (yes/no)^a^	0.0 (0, 1)	0.0 (0, 1)	0.0746
Adverse effects (yes/no)^a^			
Acne flare‐up	1.0 (0, 1)	1.0 (0, 1)	0.8210
Dryness	0.0 (0, 1)	0.0 (0, 1)	0.3631

Abbreviations: FU = follow‐up, IGA = investigator global assessment scale, Tx = treatments, (0 = clear, 1 = almost clear, 2 = mild severity, 3 = moderate severity, 4 = severe).

^a^ Yes = 1, no = 0.

**FIGURE 3:**
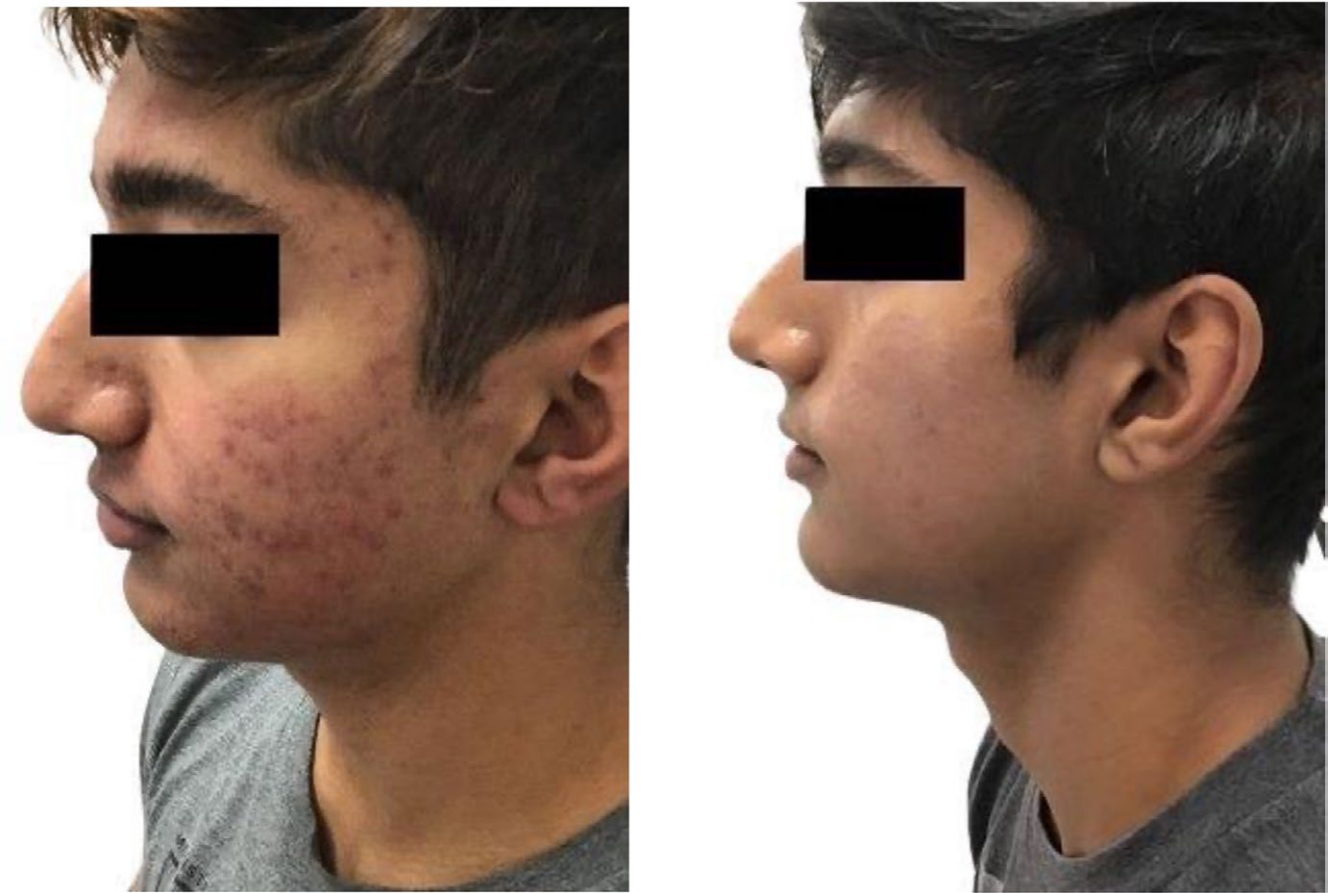
A 21‐year‐old male of Fitzpatrick skin type IV before (left) and after four treatments (right) with the 650‐ms, 1064‐nm laser, topical BPO, adapalene, and oral doxycycline. Reproduced with permission from Imahiyerobo‐Ip J, Petty A, Conza, A. How I Do it–Treating Acne and Acne Scarring in all Skin Types. *The PMFA Journal*. 2022; 10 (4): 22.

**FIGURE 4:**
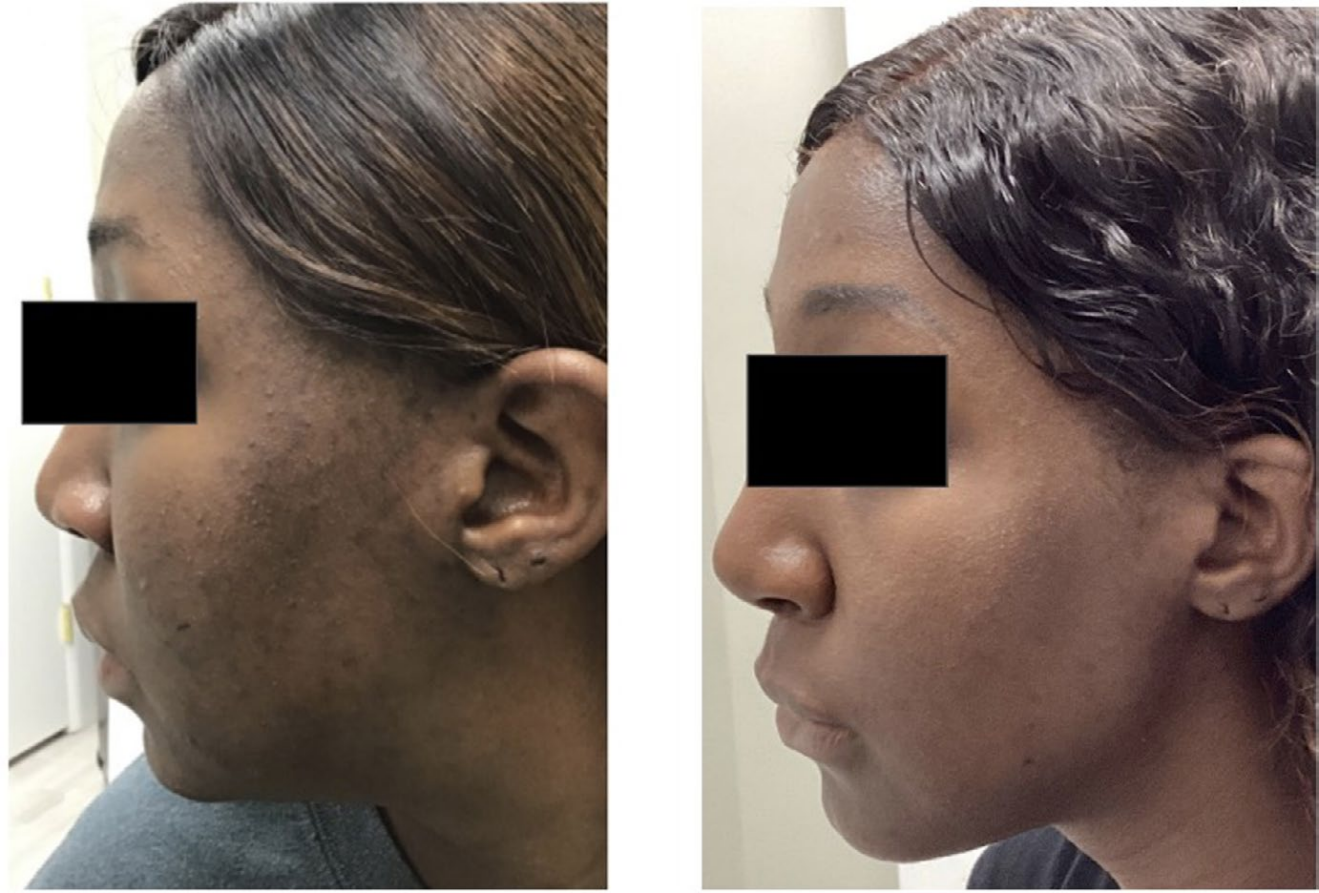
A 28‐year‐old female of Fitzpatrick skin type VI before (left) and after five treatments with the 650‐ms, 1064‐nm laser, topical azelaic acid and tazarotene (right).

**FIGURE 5:**
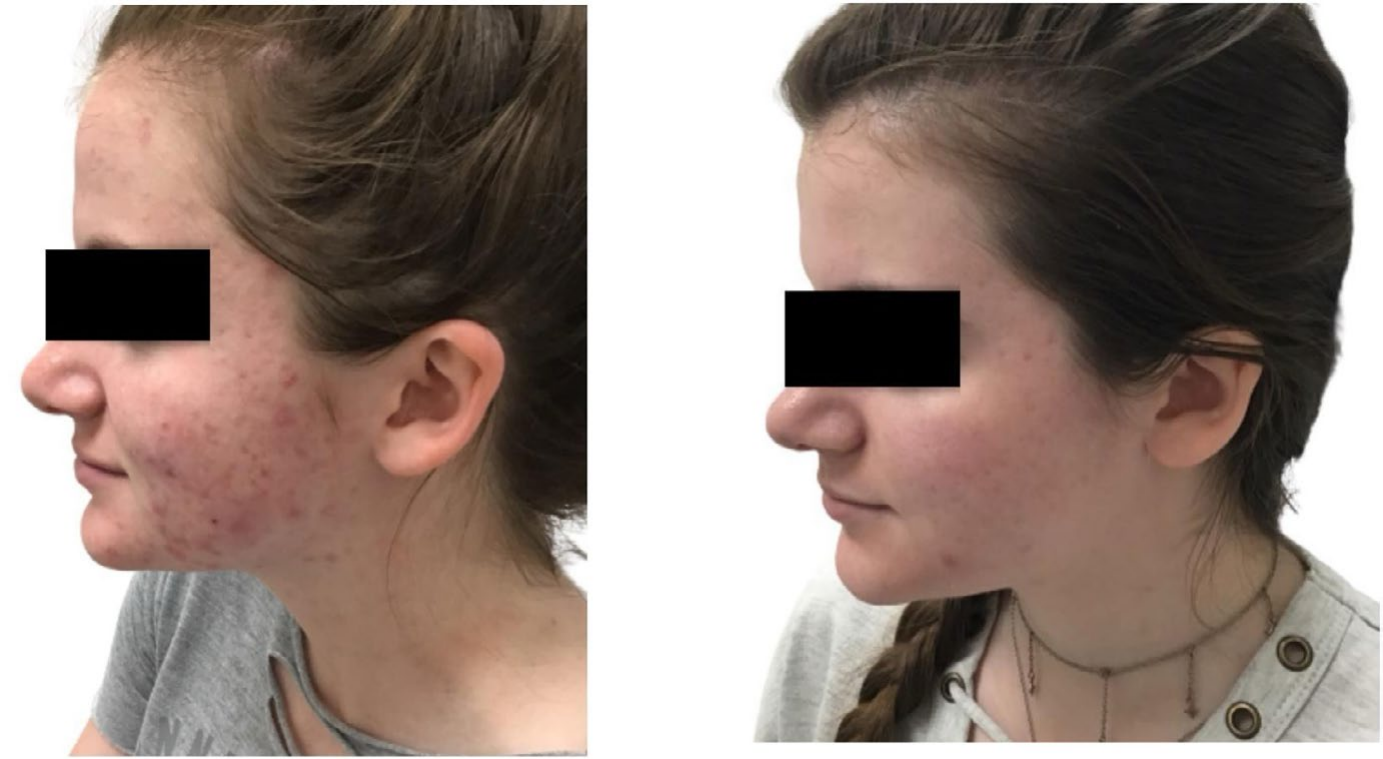
A 16‐year‐old female of Fitzpatrick skin type I before (left) and after four treatments with the 650‐ms, 1064‐nm laser, topical BPO, adapalene, tazarotene, and oral doxycycline (right).

**FIGURE 6:**
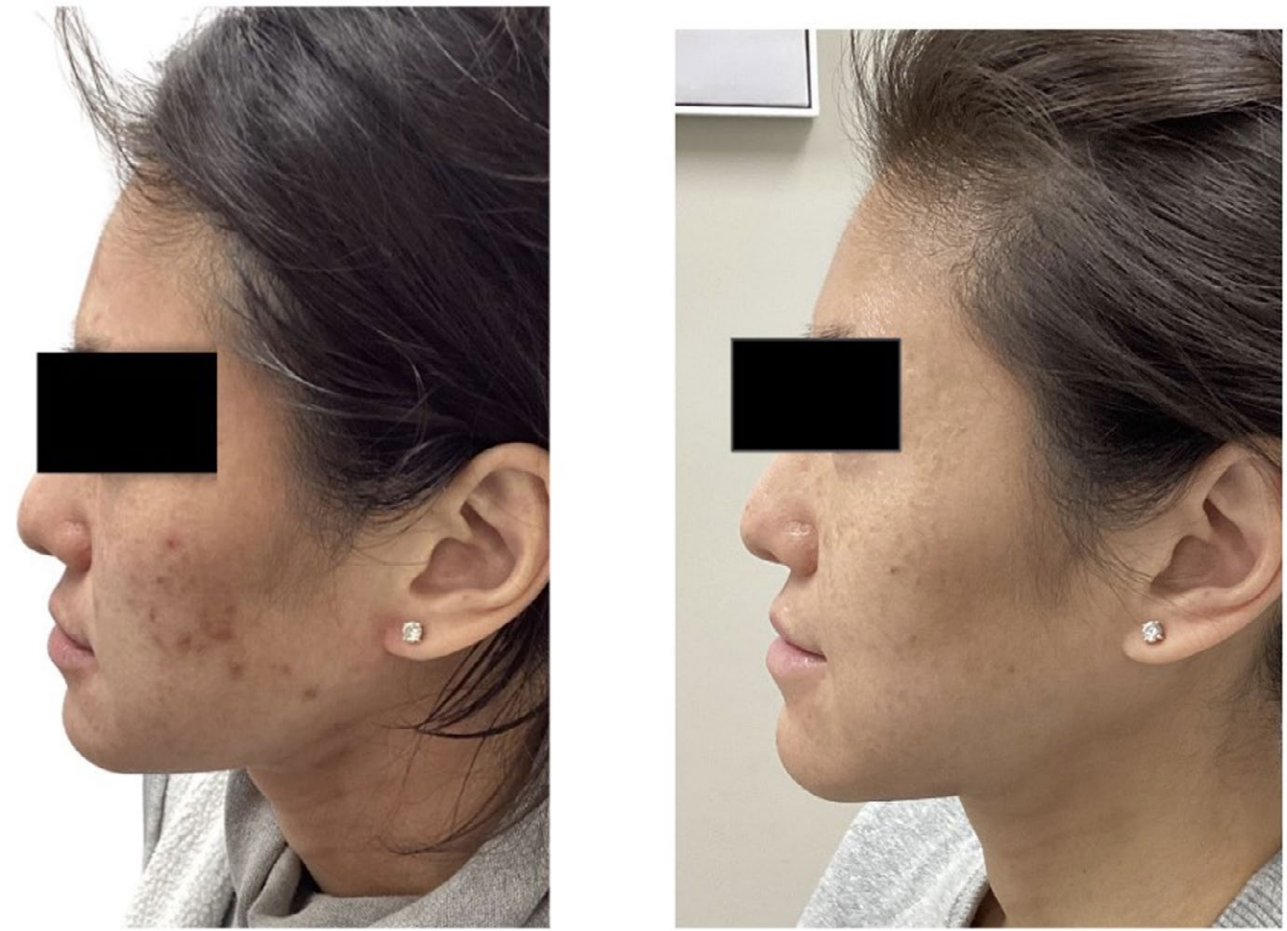
A 45‐year‐old female of Fitzpatrick skin type III before (left) and after four treatments with the 650‐ms, 1064‐nm laser and topical BPO, tacrolimus, trifarotene, and oral doxycycline (right).

**FIGURE 7:**
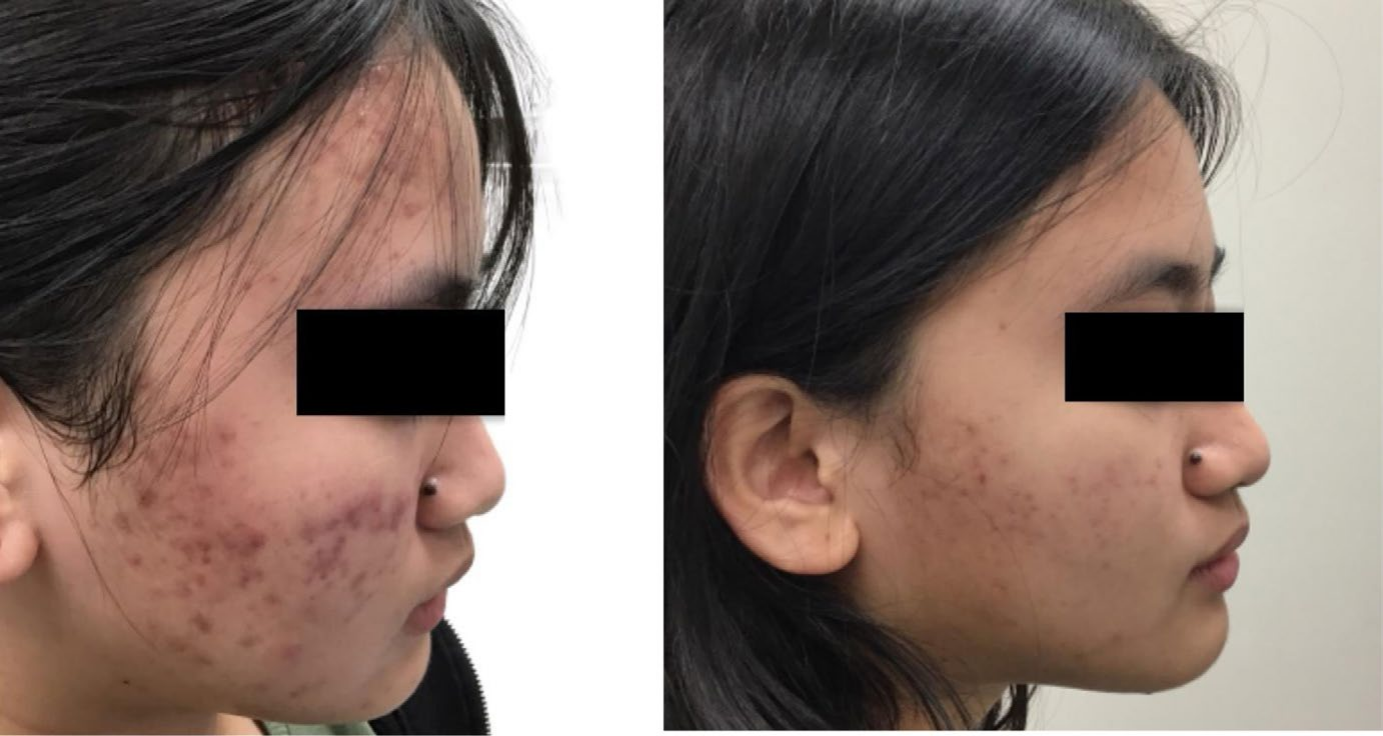
A 17‐year‐old female of Fitzpatrick skin type II before (left) and after four treatments with the 650‐ms, 1064‐nm laser, topical BPO, adapalene, minocycline, and oral doxycycline (right).

Clinical examples of patients treated with the 650‐ms laser are shown in Figures 3, 4, 5, 6, 7.

## 4 Discussion

The efficacies of energy‐based devices for the treatment of acne have been reviewed [5, 7] and are summarized in Table 9. As the table shows, a variety of lasers have been used alone and in combination to treat acne. Blue light [10, 11, 12], red light [28], red and blue light in combination [8, 9] and intense pulsed light (IPL) [13, 14], for example, have all be used to treat acne. These devices target and damage key chromophores such as hemoglobin, melanin, porphyrins produced by *C. acnes*, and water. The photoactivation of these chromophores reduces sebum production and inflammation, destroys *C. acnes*, and increases collagen production [29].

**TABLE 9:** Results of energy‐based modalities for the treatment of acne vulgaris.

Source of energy	Results	Reference
Red + Blue In combination	76% improvement in inflammatory lesions, 50% reduction in non‐inflammatory lesions (mild to moderate acne)	[8]
Red, blue alternating	81% reduction in mean lesion count	[9]
Blue light	55% reduction in acne lesions of all types	[10]
	55% reduction in lesion counts	[11]
	36% reduction in pustules and papules	[12]
IPL (430–1100 nm)	74% clearance of inflammatory acne lesions, 79% clearance of noninflammatory lesions	[13]
IPL (400–700 nm, 870–1200 nm	Inflammatory lesions reduced to 11.7% of pretreatment values, noninflammatory lesions reduced to 12.9% of pretreatment value	[14]
PDL (585 nm)	53% reduction in lesion counts	[15]
KTP (532 nm)	60%–70% clearing of acne	[16]
	60%–70% clearing of mild to moderate acne	[17]
1320‐nm Er:YAG	57% decrease in inflammatory acne lesions, 35% reduction in non‐inflammatory lesions, 30% decrease in skin sebum levels	[18]
1450‐nm	80% decrease in inflammatory lesions	[19]
	53.5% reduction in total lesion counts	[20]
	75.1% reduction in lesion counts	[21]
	67% reduction in lesion counts	[22]
	63% reduction in lesion counts	[23]
	53.2% reduction in lesion counts	[24]
1540‐nm Er Glass	71%, 79%, 73% reduction in acne lesions at 6 months, 1 years, 2 years	[25]
1726 nm	≥ 50% reduction in active inflammatory lesions 32.6% at 4 weeks, 79.8% at 12 weeks, 87.3% at 26 weeks	[26]
650‐ms 1064‐nm	48.2%, 83.7%, 86.7% reduction in lesion count after a single treatment, at treatment 3, and at 90 days, respectively	[27]

Abbreviations: Er:YAG = erbium‐doped yttrium aluminum garnet laser, IPL = intense pulsed light, KTP = Potassium titanyl phosphate, PDL = pulsed dye laser.

Photodynamic Therapy (PDT), however, combines light energy with photosensitizing agent (aminolevulinic acid or methyl aminolevulinic acid), focusing on pilosebaceous units and *C. acnes*, thus taking advantage of the bacterium's production of photosensitizing porphyrins. This modality has considerable supporting evidence that includes 13 randomized clinical trials with 701 participants. PDT is typically used to treat patients with severe acne or acne that resists other treatments [30].

Light devices also have clinical disadvantages. Blue and red light, for example, are frequently combined due to their complementary antibacterial and anti‐inflammatory properties, as well as deeper penetration of red light, resulting in a synergistic therapeutic effect [5, 31]. The disadvantage is that red and blue light therapy often requires multiple treatment sessions for optimal results [32]. PDT is limited by pain, erythema, and hyperpigmentation in patients with Fitzpatrick skin types III through VI [29, 33].

Canavan and colleagues [34] proposed that the efficacy of 400–1200 nm IPL might be due to its longer wavelengths because 1200 nm is an absorption peak of sebum. However, long‐term follow‐up data for IPL have not been reported and, for acne, this is important since *C. acnes* levels remain low only if light treatments are given over longer time periods, as with antibiotics [35]. Disadvantages of IPL include immediate erythema, burning, pain during treatment (with and without anesthesia), stinging, bulla formation, crusting, and hyperpigmentation [36].

Both the pulsed dye laser (PDL) and potassium titanyl phosphate (KTP) laser reduce levels of *C. acnes* by exciting light‐sensitive porphryins [37]. Although patient numbers are small, and long‐term treatment data are lacking for both modalities [15, 16, 17], the combination of PDL with PDT appears to be safe and effective for refractory inflammatory, comedonal, and cystic acne [38].

Mid‐infrared lasers (1320, 1450, 1540 nm) are classified as lasers that destroy sebaceous glands because they target the depth of skin where the sebaceous glands reside. Each of these modalities require cooling to protect the epidermis [5]. For the 1320‐nm laser, Deng and colleagues [17] reported six treatments to achieve the results in Table 9. Although a variety of studies have reported on the use of the 1450 nm laser [19, 20, 21, 22, 24], treatment in one study [23] was accompanied by pain in areas with many inflammatory lesions and, with the 1540 Erbium glass laser, contact cooling was necessary during treatment [25]. Additionally, a randomized, split‐face, investigator‐blinded trial of facial acne [39] demonstrated that treatment with the 1450‐nm laser did not reduce the inflammatory lesion count compared with the control.

In their 104‐patient study of patients with moderate to severe acne, Alexiades and colleagues [26] concluded that after three treatments, the 1726‐nm laser was well tolerated and patients (Fitzpatrick skin types II–VI) showed progressive improvement over 26 weeks without serious adverse events.

Saedi and colleagues [27] showed that the 650‐ms, 1064‐nm laser, after 3 treatments, provided clearance for at least 90 days in patients with mild to severe acne. The treatments were well tolerated.

The 650‐ms, 1064‐nm Nd:YAG laser delivers high energy of short‐pulse duration to target photoactive chromophores in the skin for the treatment of both acne and acne scarring. The laser also suppresses sebum production and reduces inflammation by inducing thermal coagulation of capillaries that supply sebaceous glands and inflamed skin lesions, resulting in shrinkage of sebaceous glands [5]. The 650‐ms laser promotes collagenesis and remodeling by interacting with water for the reduction in acne scars and improvement in overall skin appearance [5, 33]. While previous studies have demonstrated the effectiveness of this laser in acne treatment, most have been limited to a small number of patients. To our knowledge, our study represents the largest retrospective analysis of the efficacy of the 650‐ms, 1064‐nm Nd:YAG in the treatment of mild to severe acne vulgaris and scarring in all skin types.

When treated with the 650‐ms laser employed in this study, the majority of patients required only 2–4 treatments to achieve clearance of acne lesions. Notably, 48% achieved complete clearance. IGA scores were calculated for patients at each visit, showing a reduction from a median of 3 (moderate acne) at baseline to 1 (almost clear) 6 months post‐treatment. This indicates that many subjects maintained clear skin months after completing laser therapy, demonstrating the sustained results of the treatment. It also shows that the 650‐ms laser can be used in conjunction with various oral and topical medications, as well as non‐laser procedures (e.g, chemical peels), without adverse events.

A major advantage of the 650‐ms laser is its safety and efficacy in all skin types. This may be understood by considering the 650‐ms pulse duration, which is shorter than the thermal relaxation time of skin tissue. This reduces the risk of pigmentary changes and scarring, making it a safe choice for skin of color [40, 41]. Statistical analysis of white and dark skin showed that the number of treatments to clearance, 6‐month post‐treatment IGA scores, need for isotretinoin, and adverse events did not differ significantly between the two groups, thus expanding treatment options for skin of color.

The overall results demonstrate the viability of the studied laser device as an alternative or adjunctive therapy for acne. It shows effectiveness both with and without standard‐of‐care therapies, offering high tolerability and a favorable safety profile for all skin types. Considering these factors, high compliance is anticipated, addressing a key concern with many therapies.

Limitations of the present study include the absence of a control group and its retrospective design. The lack of insurance coverage for the present procedure may introduce selection bias because the study included only patients that could afford the procedure.

## 5 Conclusion

The 650‐ms, 1064‐nm Nd:YAG laser provides a safe and efficacious treatment of mild to severe acne in patients with white skin and skin of color.

## Author Contributions

Joyce Imahiyerobo‐Ip and Anna C. Petty were involved in the conceptualization and design of the study. Joyce Imahiyerobo‐Ip and Anna C. Petty were responsible for study execution and completion. Anna C. Petty and Idowu D. Olugbade were responsible for data analysis. Joyce Imahiyerobo‐Ip, Idowu D. Olugbade, and Anna C. Petty were involved in writing the original draft, reviewing, and editing. Joyce Imahiyerobo‐Ip, Idowu D. Olugbade, and Anna C. Petty were involved in the final review and approval of the manuscript.

## Ethics Statement

The collection and evaluation of all protected patient health information were performed in a HIPAA‐compliant manner. General study informed consent and photo informed consent were obtained before performing study procedures and taking photographs. Permission for publication was also ascertained during the informed consent process.

## Conflicts of Interest

Dr. Joyce Imahiyerobo‐Ip is a consultant for Aerolase, and she has received no additional funding for the study or preparation of this manuscript.

## Data Availability

The data that support the findings of this study are available from the corresponding author upon reasonable request.
